# Developing an efficient method for melanoma detection using CNN techniques

**DOI:** 10.1186/s43046-024-00210-w

**Published:** 2024-02-26

**Authors:** Devika Moturi, Ravi Kishan Surapaneni, Venkata Sai Geethika Avanigadda

**Affiliations:** Department of Computer Science and Engineering, Velagapudi Ramakrishna Siddhartha Engineering College, Vijayawada, India

**Keywords:** Skin cancer detection, Melanoma, Deep learning, MobileNetV2, HAM10000, Customized CNN, Flask

## Abstract

**Background:**

More and more genetic and metabolic abnormalities are now known to cause cancer, which is typically deadly. Any bodily part may become infected by cancerous cells, which can be fatal. Skin cancer is one of the most prevalent types of cancer, and its prevalence is rising across the globe. Squamous and basal cell carcinomas, as well as melanoma, which is clinically aggressive and causes the majority of deaths, are the primary subtypes of skin cancer. Screening for skin cancer is therefore essential.

**Methods:**

The best way to quickly and precisely detect skin cancer is by using deep learning techniques. In this research deep learning techniques like MobileNetv2 and Dense net will be used for detecting or identifying two main kinds of tumors malignant and benign. For this research HAM10000 dataset is considered. This dataset consists of 10,000 skin lesion images and the disease comprises nonmelanocytic and melanocytic tumors. These two techniques can be used for detecting the malignant and benign. All these methods are compared and then a result can be inferred from their performance.

**Results:**

After the model evaluation, the accuracy for the MobileNetV2 was 85% and customized CNN was 95%. A web application has been developed with the Python framework that provides a graphical user interface with the best-trained model. The graphical user interface allows the user to enter the patient details and upload the lesion image. The image will be classified with the appropriate trained model which can predict whether the uploaded image is cancerous or non-cancerous. This web application also displays the percentage of cancer affected.

**Conclusion:**

As per the comparisons between the two techniques customized CNN gives higher accuracy for the detection of melanoma.

## Background

Cancer is the term for the uncontrollable growth of tissues in a particular bodily part [1]. Skin cancer seems to be one of the dangerous diseases that is spreading vastly and quickly around the world. Skin cancer is a condition where uncontrollable growth of abnormal skin cells occurs [2]. Early detection and precise diagnosis are crucial for determining possible cancer therapies. Melanoma is very dangerous and also it is the deadliest form of skin cancer. Just 1% of all skin cancer cases come under the category of melanoma, but according to the statement given by the American Cancer Society melanoma will have a higher death rate [3]. The cells in which melanoma develops are known as melanocytes. When healthy melanocytes begin to multiply uncontrollably and create a malignant growth, the condition starts. It typically develops on sun-exposing regions of the body, like lips, hands, neck, and face. These types of cancers can only be treated if detected as early as possible they would propagate to several body parts otherwise and cause the victim to suffer a painful death [4].

Computer-aided design can be used for identifying and diagnosing cancer diseases. CAD can also be used for detecting advanced tumor disease in a cost-effective manner. By including various types of imaging techniques, the detection of cancers can be assessed. Evaluating and analyzing it is a more time-consuming process and causes error-prone. Mainly because the skin lesion images are very complicated.

The classification of skin lesions has also been focused by using machine learning techniques. The automatic skin lesion classification helps physicians to enable fast access to identifying the cancer. Machine learning can be used only when there are expert people, and it is a very time-consuming process for the selection of adequate features. At the start of the preprocessing steps, the loss of data will take place which may reduce the quality of classification. By considering the sample example a poor segmentation outcome frequently results in a poor feature extraction outcome and, as a result, a low categorization accuracy.

The most effective way to detect skin cancer can be done by using Deep Learning techniques. Deep Learning is one of the subfields of machine learning by including artificial neural network algorithms. Deep learning is majorly used in various types of domains. In deep learning, preprocessing and classification are the major components to be considered. In the preprocessing phase, the intensity of the image can be increased by removing the inconsistencies among images. In this procedure, the image will be scaled to fit into the required training model. Mostly more medical professionals have been effectively using deep learning techniques to obtain phenomenal results in many challenging situations. The layers in the various deep learning techniques rely on the classification of pixel by pixel from the lesion images. Deep learning can be used to analyze large-scale datasets more effectively and efficiently. In some situations, these algorithms may give wrong classification results. First, the broad application of various deep learning techniques for skin lesion cancer classification has been hampered by data imbalance and based upon the large volume of labeled images in the dataset [5]. These algorithms frequently lead to misdiagnosis when used to identify skin cancers that are uncommon in the training dataset [6]. Furthermore, when working with high-resolution images (like pathological images with millions of pixels), deep learning models frequently result in substantial computing costs and additional training time [7]. Additionally, due to the varied circumstances, various sounds in the picture will be produced. As a result, these methods’ robustness and generalizability should also be considered [8]. So, the appropriate model for deep learning should be selected based on the size of the dataset.

Xinrong Lu et al. [9] proposed a model for melanoma detection based on Xception Net in convolutional neural networks. In this, they suggested a system for detecting skin cancer on the basis of dermoscopic pictures. The suggested model is based on an upgraded version of XceptionNet that made use of depth-wise separable convolutions and swish activation functions. Comparing this system to the original Xception and other dome designs, the network’s classification accuracy is shown to have improved. The proposed method gives better accuracy when compared to the other comparative methods. The suggested method can be implemented with the help of other simulated state-of-the-art skin cancer diagnosis methods. The cost of training XceptionNet models was very expensive. Convolutions still make it numerically inefficient. These convolutions occur across the depth but not only in spatially.

Titus J. Brinker et al. [10] proposed an artificial intelligence technique for histologic melanoma by 18 international expert pathologists. Pathologic melanoma is classified using an inevitably arbitrary integration of a number of histologic traits. They compared CNN’s ability to differentiate between melanomas and nevi. In order to train and evaluate ensembles of three individual CNNs, two experienced dermatopathologists labeled 50 individual images of melanomas and 50 individual nevi on a single hematoxylin eosin-stained whole slide image. On slides from a different collection of images, the classifiers might not perform similarly.

Rasmiranjan Mohakud et al. [11] proposed a convolutional neural network classifier with hyper-parameter optimization for skin cancer identification using the grey wolf optimization method. It is suggested to use an automatic hyper-parameter optimized convolution neural network to identify the type of skin cancer. By using an appropriate encoding technique, their strategy optimized the hyperparameters of CNN using the Grey Wolf Optimization algorithm. By contrasting the model’s performance with that of genetic algorithm-based hyper-parameter optimized CNN and particle swarm optimization on the ISIC skin lesion multi-class data set, the model’s efficacy is confirmed. It requires expensive equipment and a lot of processing capacity.

S Bharathi et al. [12] proposed a method for melanoma recognition from nevus images. The input picture is first processed so that the noise (skin lesion) is removed from the image using a median filter and is segmented using an improved K-means clustering method. A distinct feature vector is created by extracting the required textural and chromatic characteristics from the lesion. Adaptive neuro-fuzzy reasoning system (ANFIS) and feed-forward neural network are both used to separate melanoma and nevus. (FFNN). In this study, 1023 skin pictures from the DERMIS dataset, including 104 melanoma and 917 nevus images, were used. When neural network weights or parameters are unstable, the classification output is incorrect.

Sarah Haggenmuller et al. [13] proposed a convolutional neural network for the classification of skin cancer: a review of studies incorporating human experts. The study aimed to systematically analyze melanoma and evaluate their potential clinical relevance by examining three key factors: test set characteristics (holdout/out of distribution data set, composition), test setting (experimental/clinical, inclusion of metadata), and representativeness of participating clinicians. The criteria for inclusion were fulfilled by a total of 19 studies. Two dermatopathological studies used digitized histological whole slide pictures, while six of them mainly focused on classifying the clinical images. Of these, 11 CNN-based strategies focused on categorizing dermoscopic pictures. Most of the test groups were made up of holdout images that did not accurately represent the variety of patient populations and melanoma subtypes seen in real-world settings.

Pacheco et al. [14] suggested an attention-based mechanism for combining images and metadata for the classification of skin cancer in a deep-learning model. They talk about how combining image and metadata characteristics in deep learning models for skin cancer classification can be difficult. They propose a new algorithm called the metadata processing block (MetaBlock), which uses metadata to assist data categorization by improving the extracted important features that are categorized from the images through the classification pipeline. The attention-based mechanism method known as the metadata processing block (MetaBlock) uses the metadata to improve the feature maps that are extracted from images in order to improve data classification. When we are not using the meta block, a concatenation baseline should be used to achieve precision. The current techniques require several steps in sample processing and take a lot of time to identify CTCs as indicators of metastatic development.

Dyachenko et al. [15] proposed melanoma cell detection using optical clearing and spectrum imaging in whole blood samples. These were used to discover and identify CTCs. This method was validated using imaging of affected melanoma cells and suspensions of mouse melanoma cells of line B16F10 alone and in combination with blood. The method of cleaning rodent blood optically with biocompatible chemical agents was used to show a technique for increasing detection. The results indicate that the proposed diagnostic method has the ability to detect CTCs in whole blood samples from melanoma patients quickly. This methodology works only by considering the single cancer type cells and also when identifying the melanocytic cells in the blood layer it decreases the blood scattering.

Song et al. [16] proposed an end-to-end multitask deep learning framework for skin lesion analysis. The suggested method can detect, classify, and segment skin lesions all at the same time. A loss function based on the focal loss and the Jaccard distance is suggested to minimize the class imbalance problem in the dataset (which is common in medical image datasets) while also improving segmentation performance. For improving the efficiency of feature learning a phase joint strategy has been used in framework training. Training of the network gradually decreases by introducing the vanishing gradient effect.

Lisheng Wei et al. [17] proposed a technique for the detection of skin cancer in dermoscopic images by using an ensemble lightweight deep learning network. It has two standard lesion classification networks and feature discrimination network feature extraction components. The first module (Lightweight CNN) of the model receives two groups of training samples (positive and negative sample pairs). The outputs from the feature extraction module result in two sets of feature vectors which can be then used for performing the training or classifying the two networks. It provides better performance for all the tasks of segmentation as it operates with a smaller number of training samples. Learning may decrease in the middle layers, as there is a possibility that network learning will ignore the layers where hided features are indicated.

Abder-Rahman H. Ali et al. [18] proposed the rule known as the ABCD rule for melanoma detection. This method can be useful for automatically identifying dermoscopic images through a pipeline of stages like segmentation, feature extraction, and classification. Color variation majorly describes the greater number of shades that are present with the border of the skin lesion image. Generally, the melanoma lesions consist of two or more colors whereas the benign lesions consist of uniform colors. Combining the features of ABCD (AB, AC, AD, BC) has the greater possibility of identifying the skin lesion images easily. Using various machine learning approaches like SVM helps to identify or classify the lesions in either a symmetrical or asymmetric manner. In order to get more accuracy for the asymmetry various metrics have been merged into a single vector. This approach is less used because some dermoscopic images have different noises like lightening changes and bubble or hair occlusion.

## Methods

### Study design

**Fig. 1:**
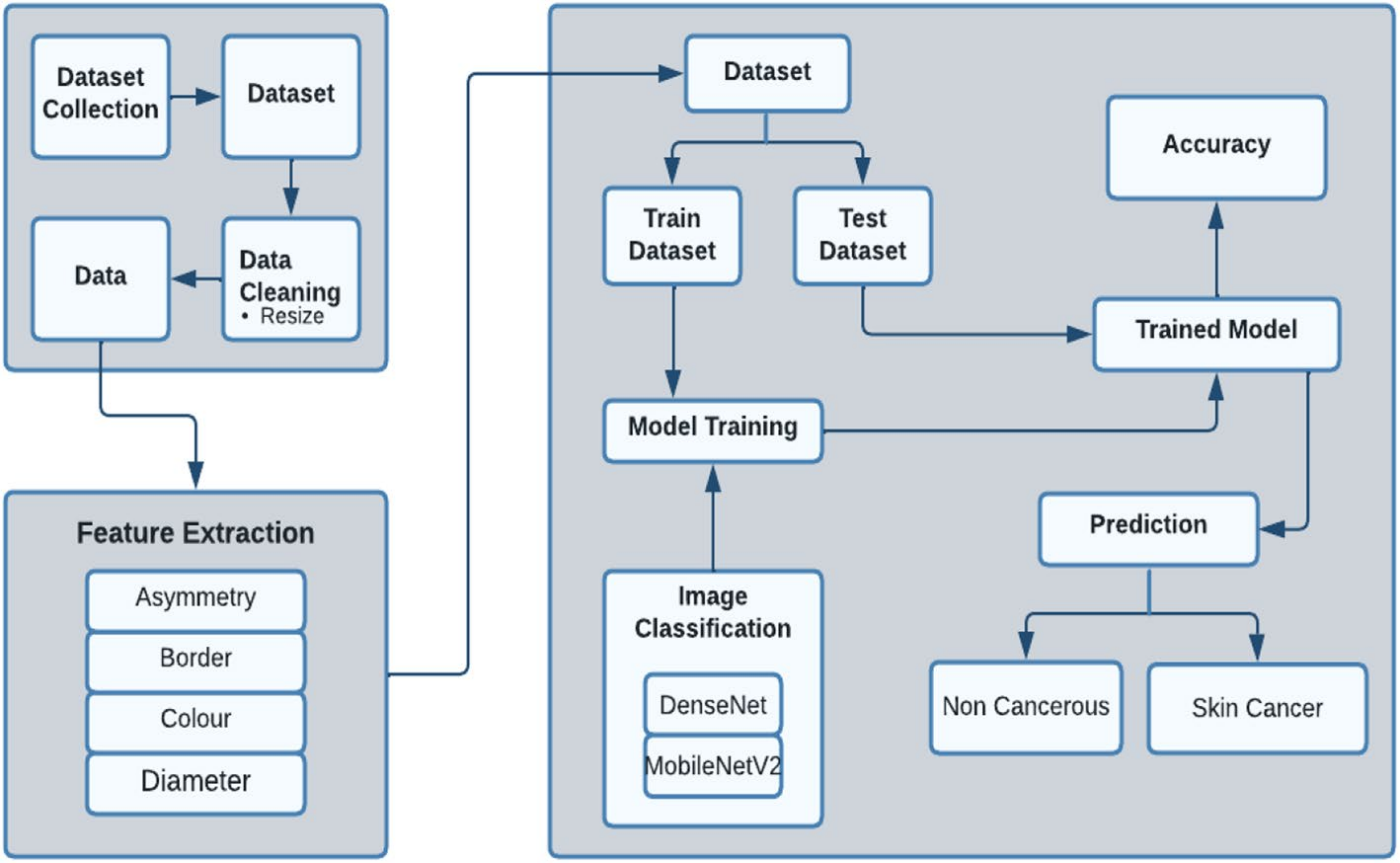
Proposed methodology

In this project, the performance analysis is made between mobilenetv2 architecture and dense net architecture. The main goal is to identify the best architecture that has been trained over HAM10000 dataset. Figure 1 depicts the proposed system of this research. Our project can be divided into modules like dataset collection, data pre-processing, feature extraction, partitioning of data into training and testing datasets, training the model using dense net and mobilenetv2 architectures, and predicting the skin lesion type. Finally, A Flask-based UI has been developed that is integrated with the best-trained model.

### Data collection

**Fig. 2:**
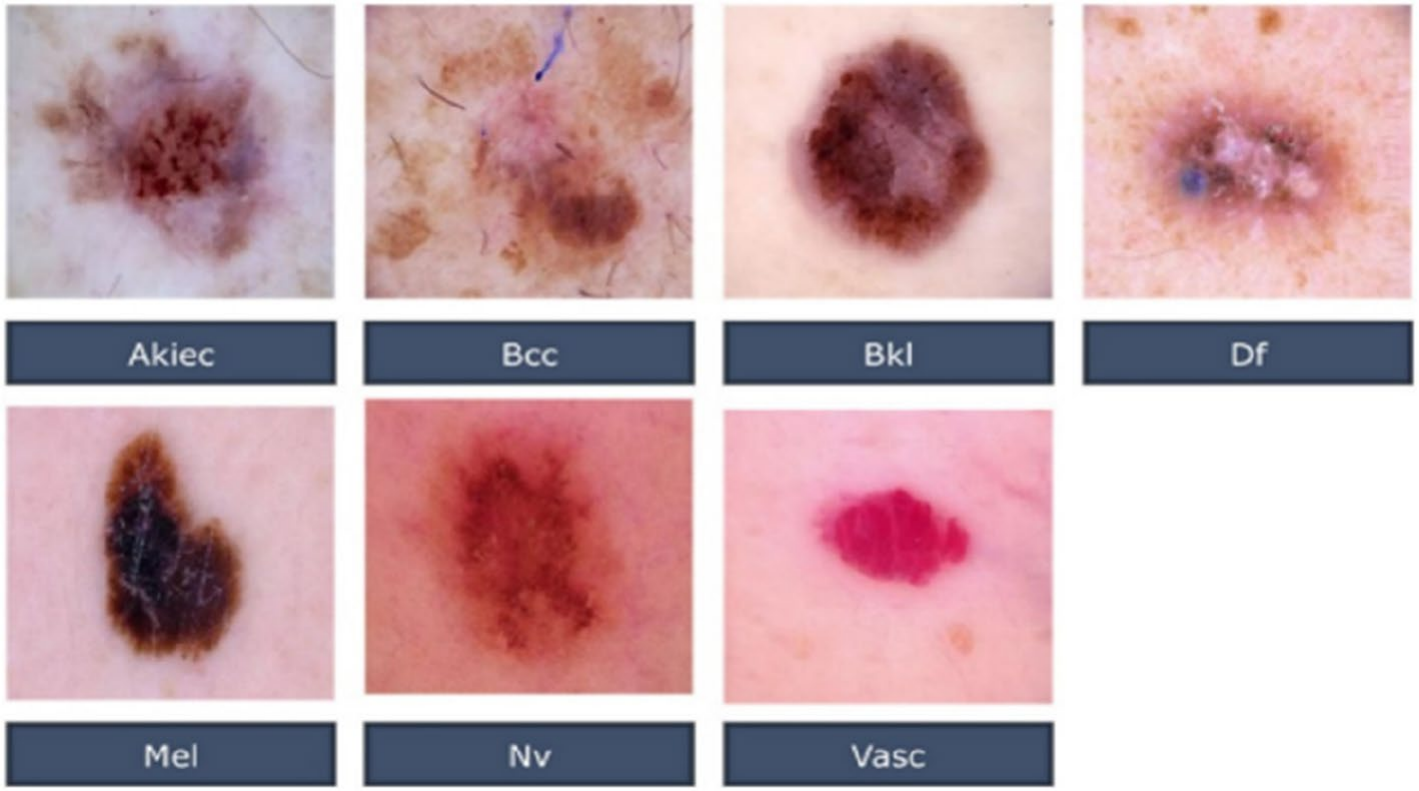
Types of diseases in dataset

The dataset used in this methodology is HAM1000 dataset. HAM10000 stands for Human Against Machine with 10,015 images. The dermoscopic images in the dataset consist of 10,015 images with 7 classes. These are collected from different populations and gathered and stored using different modalities. The dataset has been directly used from the Kaggle [19]. The classes are Actinic keratoses and intraepithelial carcinoma or Bowen’s disease (akiec), basal cell carcinoma (bcc), benign keratosis-like lesions (bkl), dermatofibroma (df), melanoma (mel), melanocytic nevi (nv) and vascular lesions (vasc) which are considered for skin lesion image classification. The below images describe the different types of lesions in the dataset Fig. 2.

**Fig. 3:**
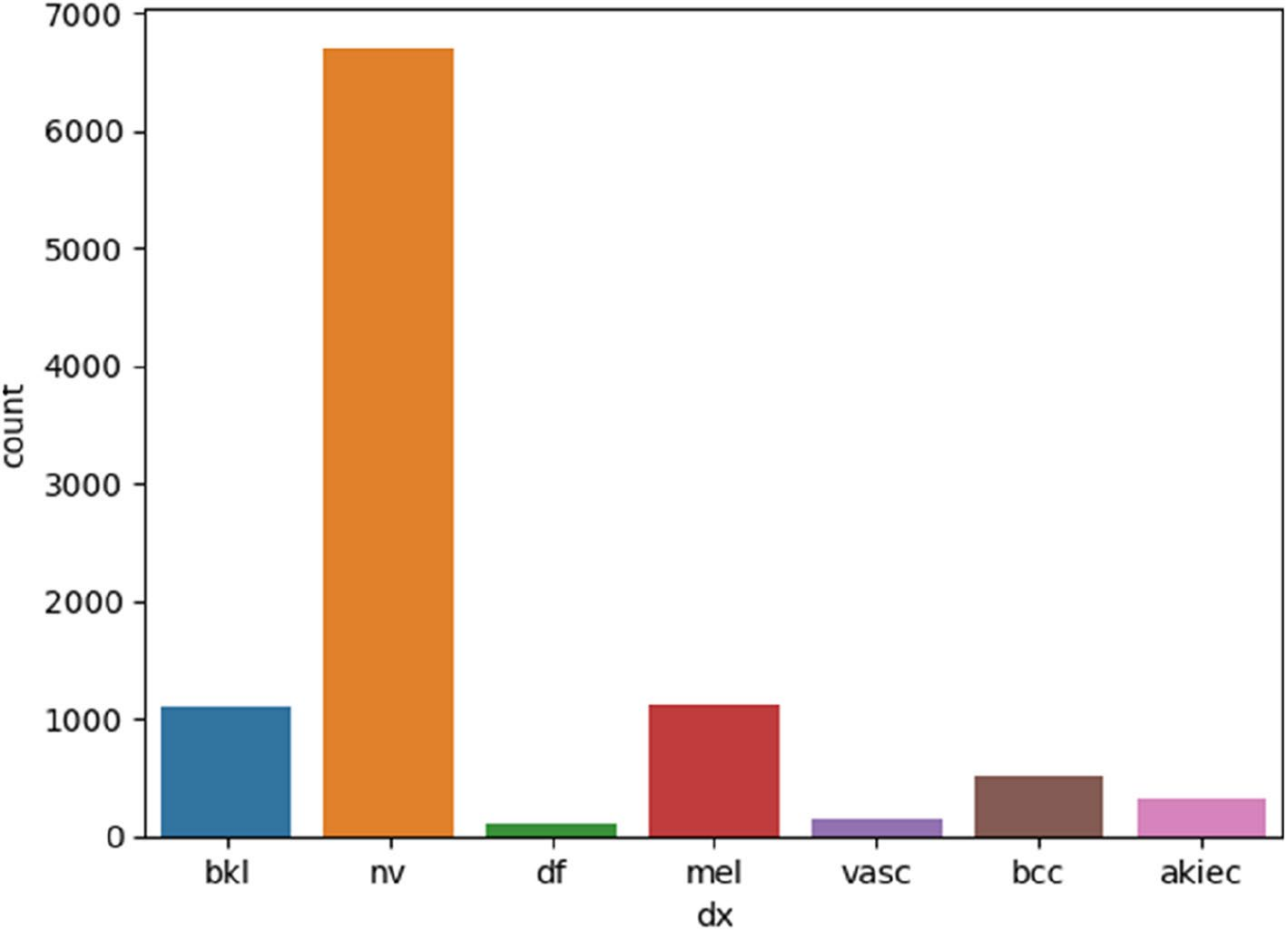
Exploratory data analysis

There are various attributes present in the data set that help to classify the disease. The various attributes present in the dataset are lesion id, image id, dx, dx type, age, sex, and localization. dx stands for the type of the disease that has been affected. localization indicates the place where the disease has been affected. The dataset can be visualized by plotting image count against lesion id as shown in Fig. 3.

### Preprocessing

The complete dataset is made up of different types of skin lesion images. In this instance, pre-processing is carried out to match the specified raw size input into the input layer’s image. Also, we can pre-process the data to enhance its characteristics and the effectiveness of the model. Pre-processing will be used to identify the various types of abnormalities present in the image. Choosing the best pre-processing technique helps to increase the accuracy and classification. The stages that are included in the pre-processing are image scaling and removing hair from the lesion images. Images that are gathered from different sources will be in different sizes. Image scaling resizes the images into fixed width pixels of size and variable size of height. Hair removal should be performed before the segmentation process or else it will mislead the segmentation process. To remove the hair from skin lesion images methods like mathematical morphology are used [20]. Hence, the hair-free images will be used for further steps to perform various operations.

## Model training

### Convolutional neural networks

A particular kind of neural network is a convolutional neural network. It consists of several convolutional layers that can be used to identify or extract various features directly from the images present in the dataset. Later on, these images will be used for further classification. Convolutional neural networks have been used to improve the accuracy and performance in various kinds of applications. It is present in various architectures. Some of the architectures are MobileNetV2, and customized CNN. These architectures of the CNN will be trained on very large datasets. In this section, a brief explanation of these two architectures will be discussed to extract the features of various skin lesion images. In order to develop an efficient model first the performance analysis between the two models was discussed.

### MobileNetV2

**Fig. 4:**
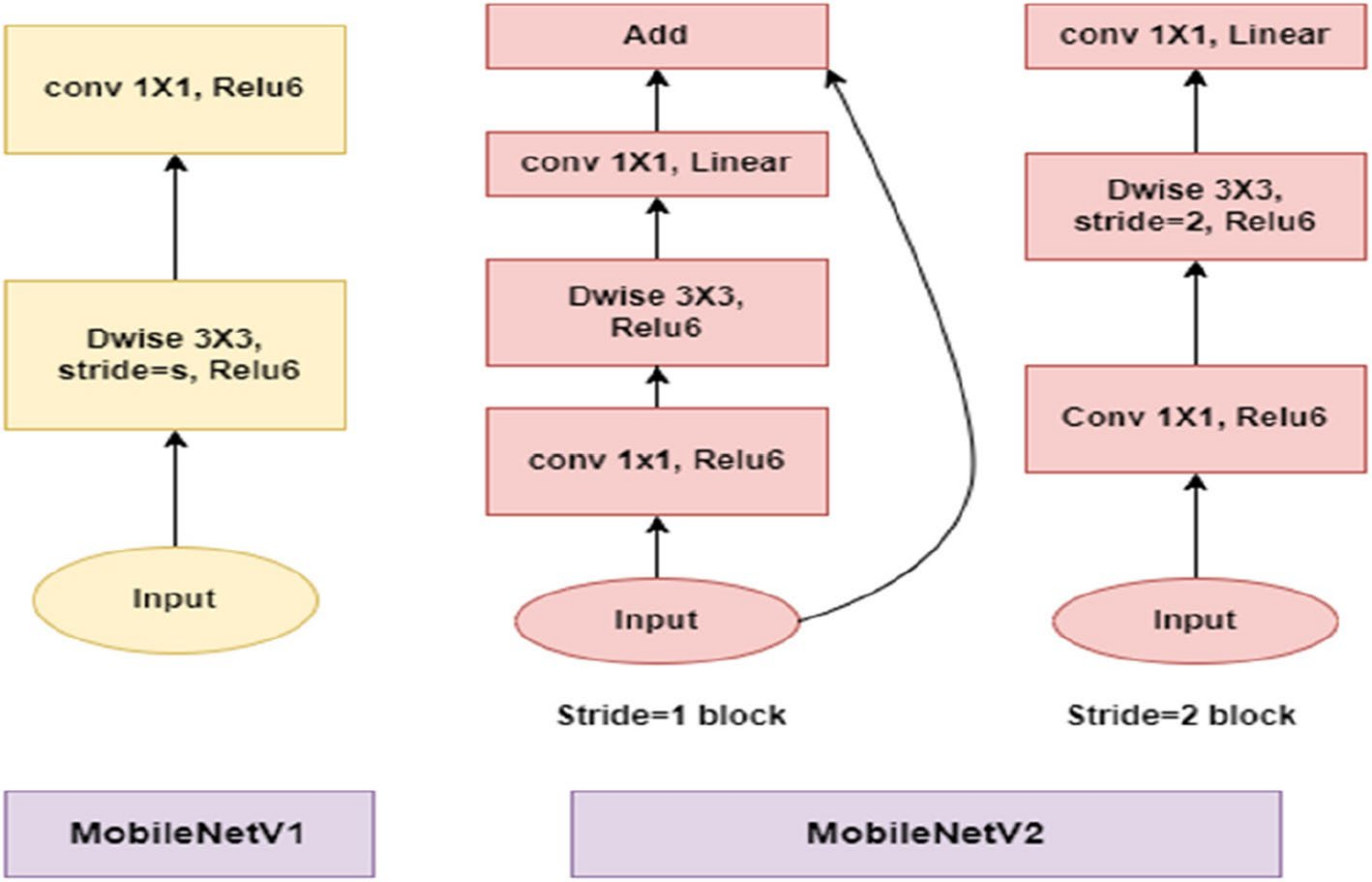
Architecture of MobileNetV2

MobileNetV2 architecture is a convolutional neural network model. To conduct the classification for the provided input image, it has 53 layers. Because of its lightweight architecture, the algorithm performs well. In order to increase accuracy, MobileNetv2 was designed using MobileNetv1 as a reference. The given inputs are filtered by the 2D convolution architecture into a single output channel. It will be transported into several channels for the output. Just 1 × 1 convolution also referred to as point-wise convolution is used to filter the stacked output channels. MobileNetV1 has 28 by separating the depth-wise and point-wise convolutions; however, MobileNetv2 has a different number. The required picture size for the MobileNetv1 and MobileNetv2 is 224 × 224 × 3. Both the classification and the object detection employ MobileNetv2 architecture (Fig. 4).

### Customized CNN

**Fig. 5:**
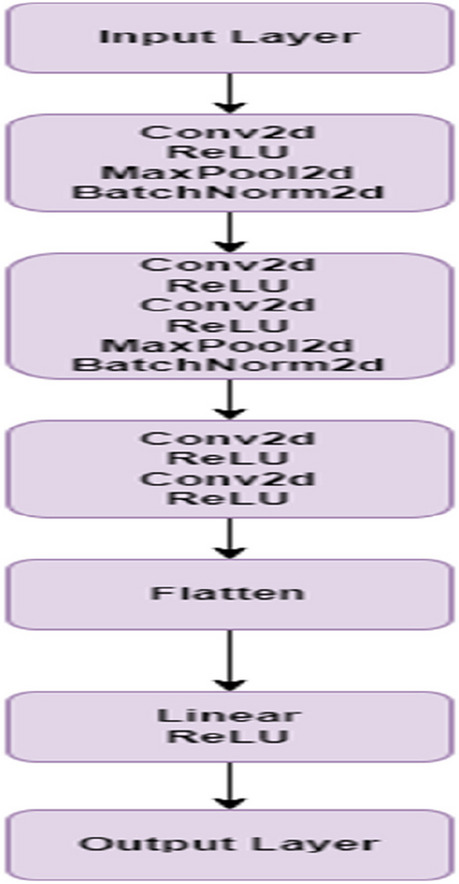
Architecture of customized CNN model

A customized CNN (convolutional neural network) is a neural network architecture that has been specifically designed and tailored for a particular task or problem. CNNs are a type of deep learning network commonly used for image recognition, classification, and segmentation tasks. Customized CNNs typically involve modifying the architecture and/or hyperparameters of a standard CNN to improve its performance on a particular task. This can involve changing the number and size of convolutional layers, adjusting the pooling and activation functions, adding regularization techniques such as dropout or batch normalization, and altering the optimizer and learning rate. The customization process often involves a combination of domain expertise and experimentation to find the optimal architecture and hyperparameters for a particular task. This can be done using techniques such as grid search, random search, or Bayesian optimization. Overall, a customized CNN can provide improved accuracy and efficiency for a specific task, compared to using a generic CNN architecture. However, it requires significant expertise and resources to design and train a customized CNN, making it a more advanced technique in the field of deep learning (Fig. 5).

**Figure:**
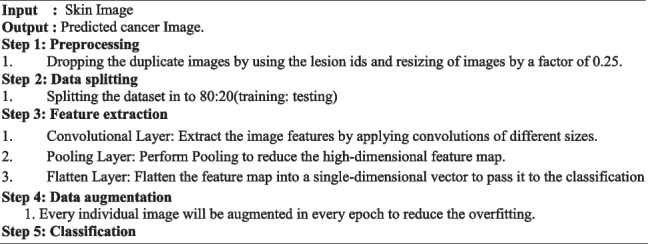
** Algorithm 1.** Skin detection using CNN techniques

### Implementation of web app

**Fig. 6:**
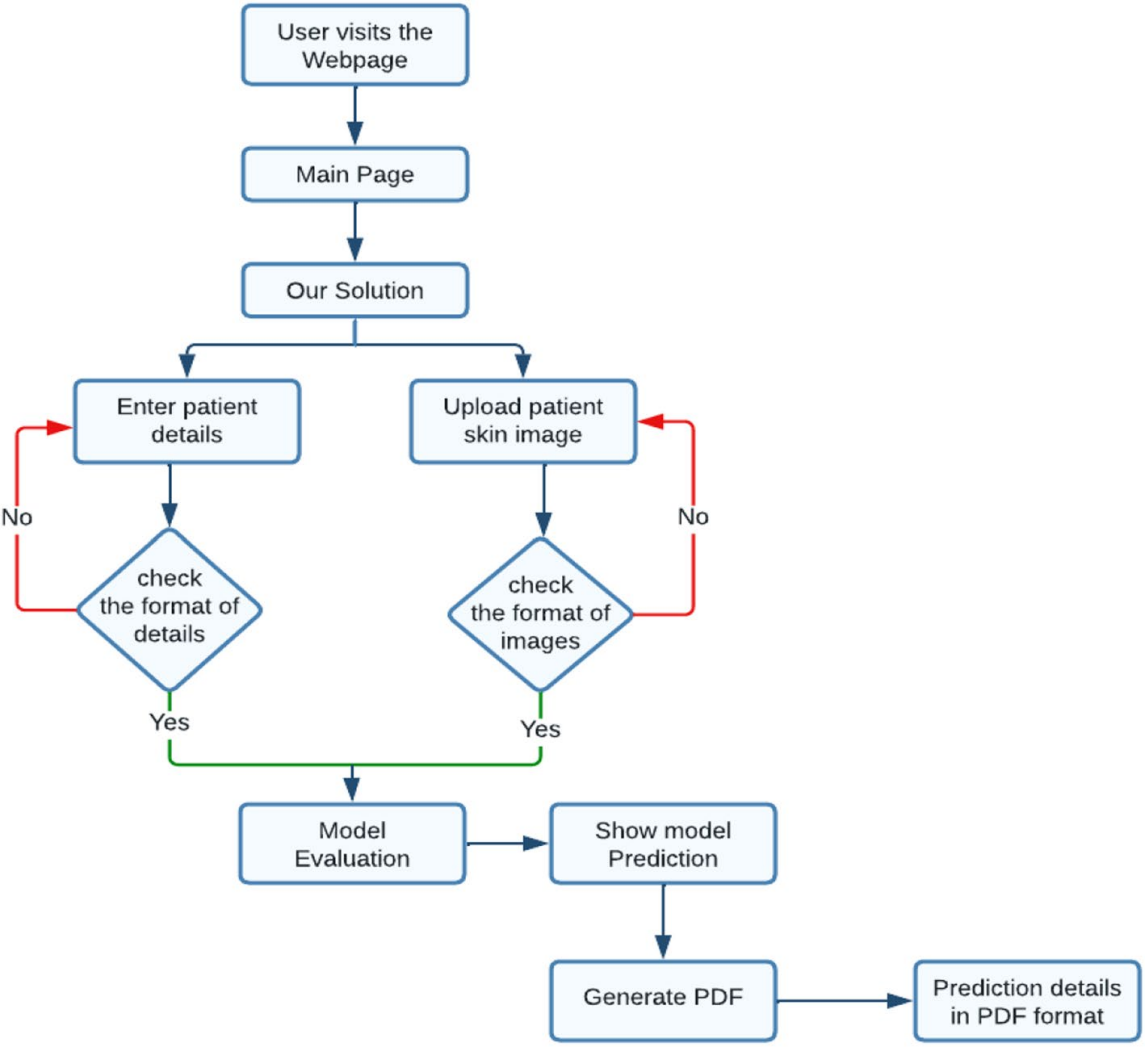
Flow chart for the web interface

The Implementation of the web user interface can be done by using the flask framework. Based on the accuracy obtained in the model training the model with higher accuracy will be selected. From the above methodology, we can conclude that the customized CNN approach gives better accuracy than MobileNetV2 (Fig. 6).

There are various steps that are involved in order to perform the prediction using a web application. The step-by-step procedure for the web application is discussed below:

- Step 1: First the user visits the webpage to perform the detection.
- Step 2: In our solution menu, it will ask the user to enter the patient details, and to upload the patient skin lesion image.
- Step 3: In this step, it will check whether the entered details and uploaded image format are accurate or not. If it is accurate then go to step 5.
- Step 4: If the entered data and uploaded image are not accurate then go to step 2.
- Step 5: Model Evaluation will take place with the help of customized CNN.
- Step 6: Displays the model prediction results with the help of plotting and the percentage it has been affected.
- Step 7: In Step 7, the user has the capability to download the entire predictions in PDF format.

### Statistical analysis

In statistical analysis, accuracy has been majorly considered. After the comparison of the two architectures, it has the customized CNN gives better accuracy than MobileNetV2. The accuracy obtained using the customized CNN is 95% and MobileNetV2 is 85%.

## Results

The two architectures that are discussed in the methodology are trained under HAM10000 dataset. The Model accuracy and Model loss results for the MobileNetV2 and customized CNN will be discussed below.

### Model accuracy and model loss results for MobileNetV2 and customized CNN

**Fig. 7:**
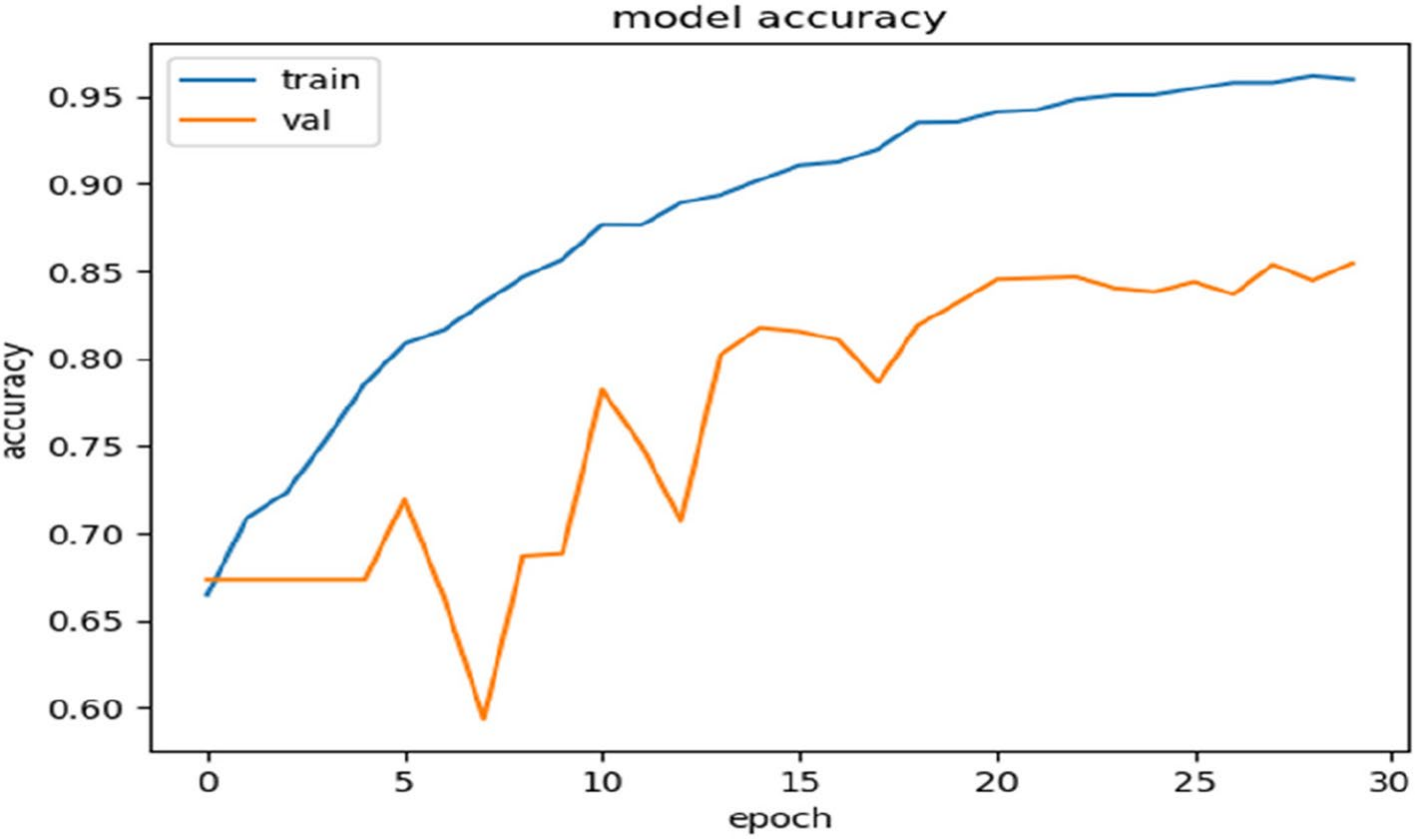
Model accuracy using MobileNetV2

**Fig. 8:**
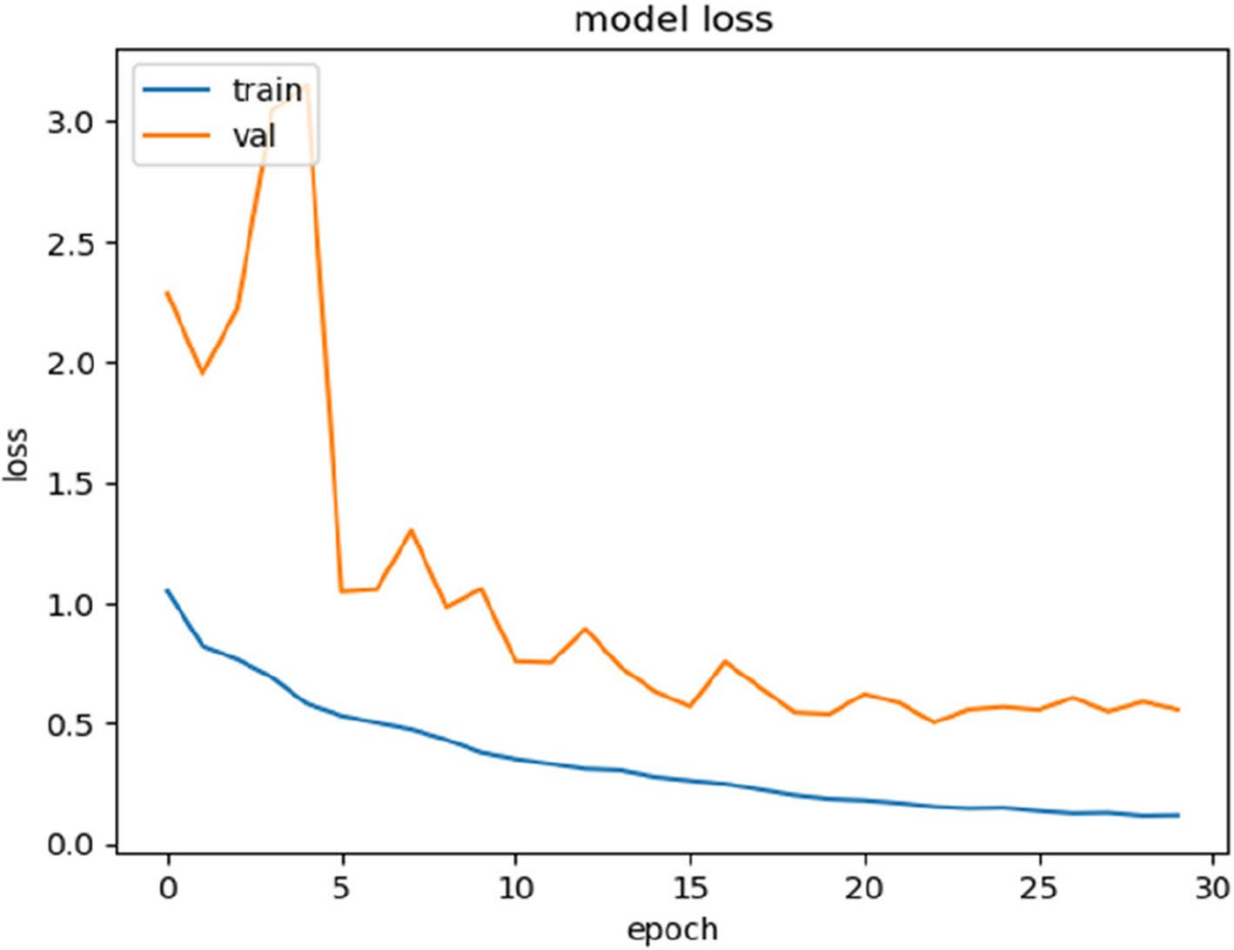
Model loss using MobileNetV2

**Fig. 9:**
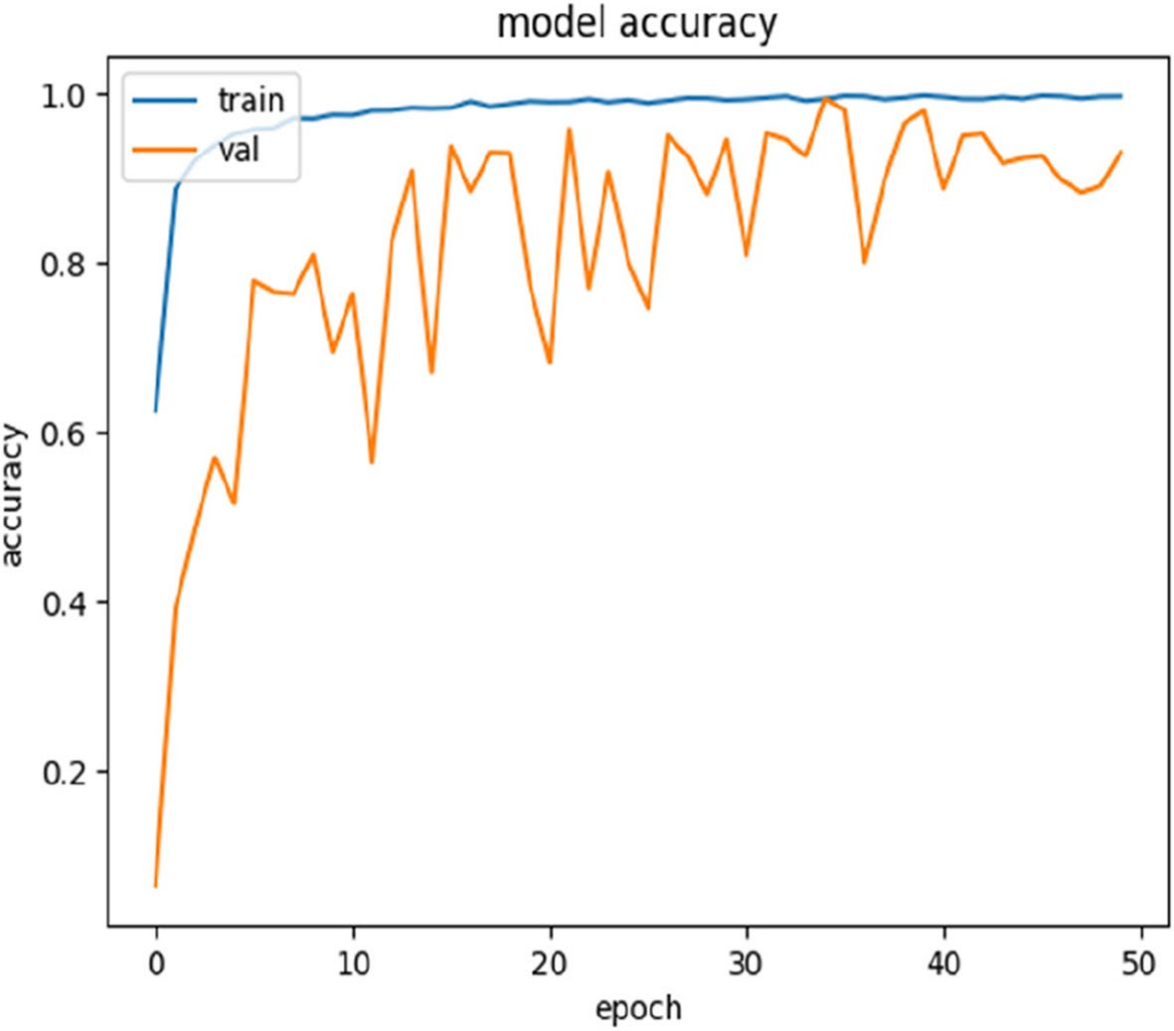
Model accuracy using customized CNN

**Fig. 10:**
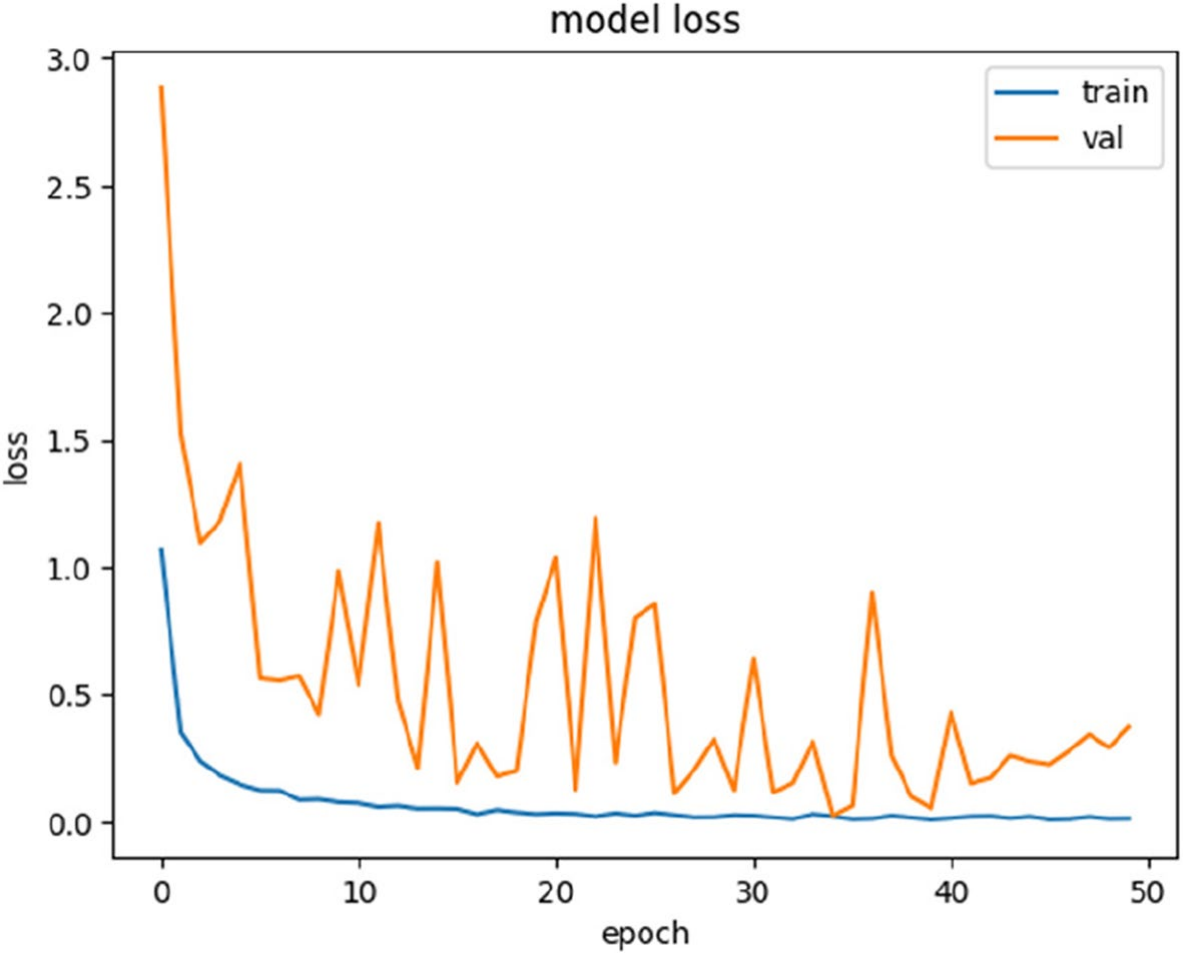
Model loss using customized CNN

Model accuracy is the metric that is used to evaluate the model’s performance. It helps us to know which model is best at identifying the relationships and links between the variables. In this, the model’s accuracy on the training dataset is determined by its training accuracy, and its accuracy on the testing dataset is determined by its validation accuracy. From our observations, mobilenetv2 model has a training accuracy of 0.98 and a validation accuracy of around 0.85. The customized CNN model has a training accuracy of around 1.0 and a validation accuracy of 0.95. Model loss helps us to compare the difference between the actual output value and the expected output value. In this, the training loss for the moblinetv2 model and customized CNN model reaches 0 and the validation loss for mobilenetv2 is 0.6 and for customized CNN is 0.45 (Figs. 7, 8, 9 and 10).

### Web application outputs

**Fig. 11:**
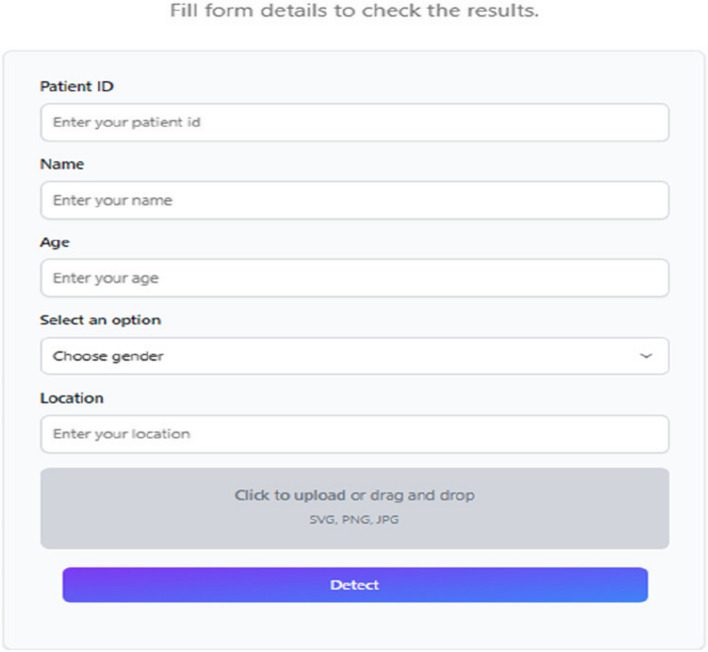
The basic UI interface allows user to enter details

**Fig. 12:**
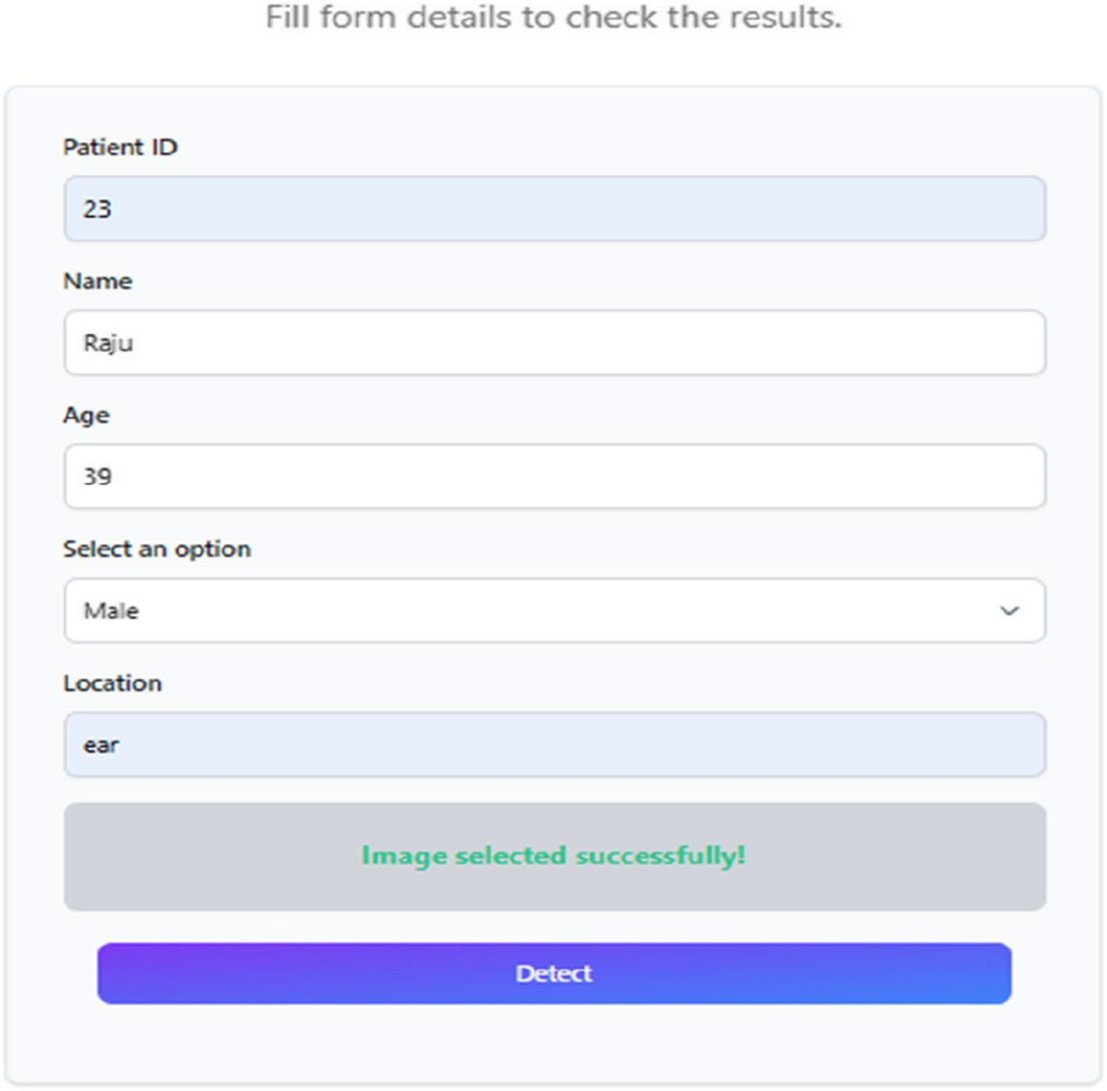
Describes details entered by the user

**Fig. 13:**
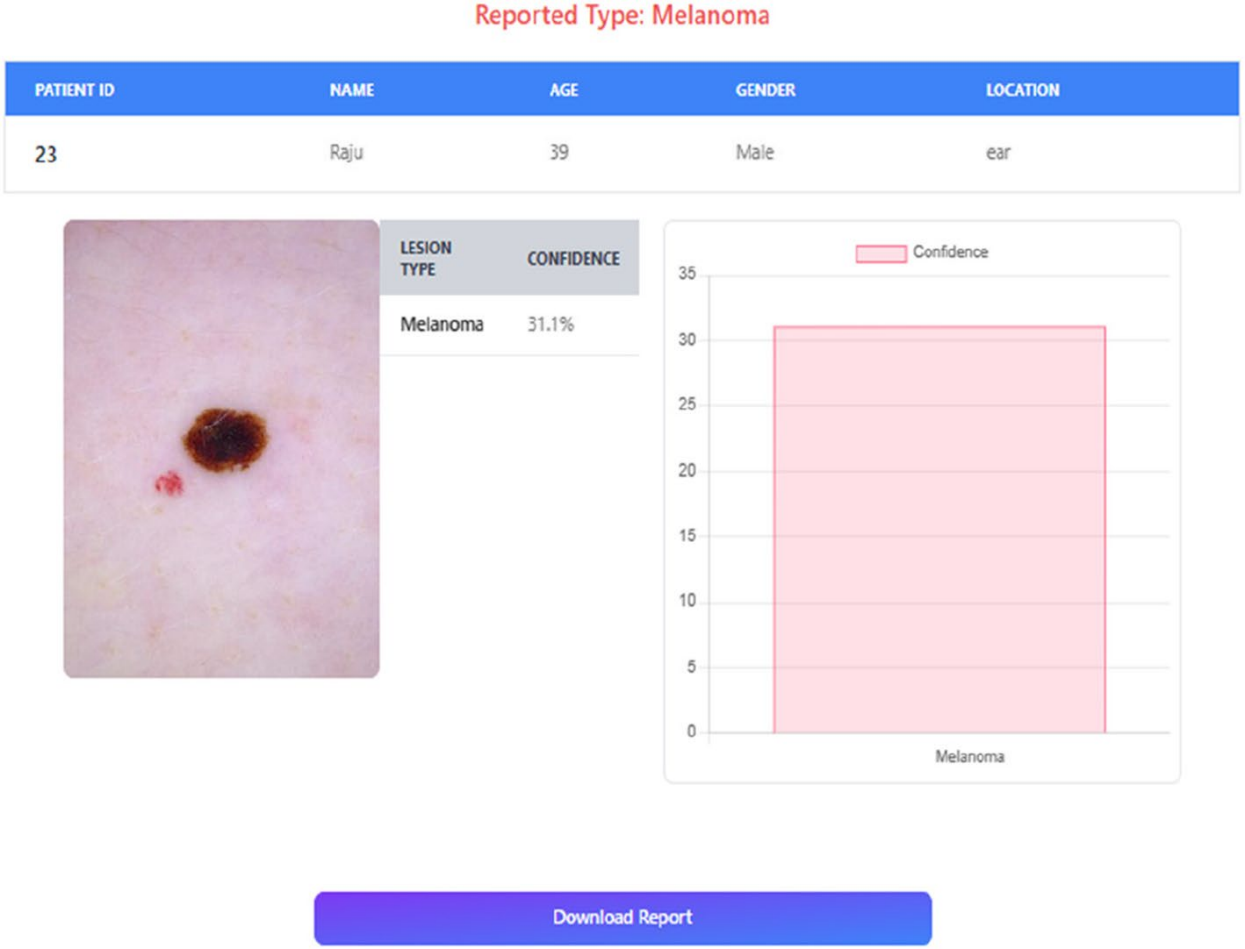
Describes the predicted output with percentage

Initially, the web interface is displayed with the following image. It asks the user to enter the corresponding details accurately (Figs. 11, 12 and 13).

## Discussion

After performing the different tests on different images in the dataset the drawback has been identified. If we try to add the new lesion image in the dataset for the classification then it gives the wrong classification result. To rectify this problem, we need to add the malignant skin lesion images of the same dataset. Two datasets will not contain the same pre-processing techniques, so this is the case to add the same lesion images in the same dataset. Image classification systems’ performance can be varied by performing various alterations in the images like rotation and flipping. If we include all these transformation techniques in the training dataset definitely the strongness of the model will be increased. To reduce the overfitting in the two architectures we have used data augmentation techniques.

Many artificial intelligence algorithms majorly focus on lesion images with more injury. The surrounding part will not be considered by these algorithms. In order to get the correct diagnosis of the image the surrounding part should also be considered. In the case of detection of melanoma, the entire area around the skin lesion image will be considered for the detection. We followed the rule ABCDE for detecting the injured and surrounding area.

## Conclusions

In this paper, the background study of two algorithms MobileNetV2 and customized CNN has been used. The architectures and the pre-processing techniques used in this research were discussed in the “Methods” section. The experimental results in terms of accuracy were discussed in the “Results” section. Based upon the model accuracy results obtained by the two models it can be concluded that customized CNN gives high accuracy than MobileNetV2. These models will be used to integrate with the flask-based GUI developed web application.

## Data Availability

The dataset generated and/or analyzed during the current study is available in the HAM10000 repository, https://www.kaggle.com/datasets/kmader/skin-cancer-mnist-ham10000.

## Abbreviations

CNN: Convolutional neural networks
HAM10000: Human Against Machine with 10000 training images
CAD: Computer-aided diagnosis
ABCDE: Asymmetry, border, color, diameter, evolving
akiec: Actinic keratoses
bcc: Basal cell carcinoma
bkl: Benign keratosis lesions
df: Dermatofibroma
mel: Melanoma
nv: Melanocytic nevi
vasc: Vascular lesions
train: Training
val: Validation
